# Evaluation of vascular pain in patients with colorectal cancer receiving peripheral venous chemotherapy with or without oxaliplatin

**DOI:** 10.1038/s41598-018-37966-w

**Published:** 2019-02-12

**Authors:** Taisuke Matsuoka, Yoichiro Yoshida, Naoya Aisu, Teppei Yamada, Ai Mogi, Akira Komono, Ryohei Sakamoto, Daibo Kojima, Gumpei Yoshimatsu, Fumiaki Kiyomi, Shohta Kodama, Suguru Hasegawa

**Affiliations:** 1 Department of Gastroenterological Surgery, Fukuoka University School of Medicine, Nanakuma 7-45-1, Jonan-ku, Fukuoka, 814-0180 Japan; 2 Division of Oncology, Hematology and Infectious Diseases, Department of Internal Medicine, Fukuoka University Faculty of Medicine, Nanakuma 7-45-1, Jonan-ku, Fukuoka, 814-0180 Japan; 3 Department of Regenerative Medicine and Transplantation, Faculty of Medicine, Fukuoka University, Nanakuma 7-45-1, Jonan-ku, Fukuoka, 814-0180 Japan; 4 Academia, Industry and Government Collaborative Research Institute of Translational Medicine for Life Innovation, Fukuoka University, Nanakuma 7-45-1, Jonan-ku, Fukuoka, 814-0180 Japan

## Abstract

Oxaliplatin is a key chemotherapy drug in patients with colorectal cancer. Administration of oxaliplatin via a peripheral vein often causes vascular pain. However, no studies have evaluated vascular pain in patients with colorectal cancer in relation to peripheral venous administration of chemotherapy with or without oxaliplatin. We evaluated oxaliplatin-induced vascular pain using subjective and objective methods. We determined if oxaliplatin induced vascular pain in patients with colorectal cancer using a Visual Analog Scale (VAS) and a PainVision PS-2100 device. We compared VAS score between chemotherapy regimens with or without oxaliplatin, and between genders. We also examined the correlations of VAS score with pain intensity examined by the PainVision PS-2100, and with age and vessel diameter. A total of 98 patients with colorectal cancer were enrolled in this study, including 78 patients who received oxaliplatin via peripheral venous administration and 20 who received chemotherapy without oxaliplatin. The median VAS scores in patients with and without oxaliplatin were 36.5 (interquartile range 9.0–60.0) and 0 (0–4.0), respectively (*P* < 0.001), and the median pain intensities according to PainVision were 43.5 (14.3–98) and 36.5 (9.3–58.5), respectively (*P* < 0.001). There was a positive correlation between VAS and pain intensity (r = 0.584), but no correlation between VAS score and age (r = −0.174) or vessel diameter (r = −0.107). Peripheral venous administration of oxaliplatin induced vascular pain, measured both subjectively and objectively, in patients with colorectal cancer, regardless of vessel diameter.

## Introduction

Oxaliplatin has been a key chemotherapy drug for the treatment of colorectal cancer (CRC) since the establishment of the FOLFOX (fluorouracil plus leucovorin and oxaliplatin) regimen^1^. CAPOX (oxaliplatin and capecitabine), as well as FOLFOX, is recommended as a first- and second-line chemotherapy for metastatic CRC, based on the phase III NO16966 trial designed to compare CAPOX with FOLFOX4^2^. However, CAPOX therapy with peripheral venous administration of oxaliplatin has been reported to cause vascular pain^3,4^, and the low pH and hyperosmolarity of the solution, as well as small vein size and rapid infusion rate, are known to be associated with infusion phlebitis^5^. The development of vascular pain and phlebitis following intravenous infusion of antineoplastic agents subsequently increases the risk of discontinuation of chemotherapy^6^. Co-infusion of dexamethasone^4,7,8^, prewarming the peripheral blood vessels^9^, premedication with oxycodone^6^, and changing the dose rate have all been investigated as ways of reducing oxaliplatin-induced vascular pain. To determine which techniques are most effective, it is necessary to objectively evaluate vascular pain. Clinical trials using objective assessment methods would lead to minimization of vascular pain and improvement in patients’ QOL. However, vascular pain has usually been evaluated by subjective methods, such as questionnaires and visual analogue scale (VAS). The VAS is the most widely used tool for evaluating pain intensity^10^. Patients were asked to indicate a point along a 100 mm scale bar depending on the strength of their pain, with ‘no pain at all’ (0) at the left end of the scale and the worst pain (100) at the right end of the scale bar. The VAS score is considered to be a good indicator of subjective pain. Few studies have assessed oxaliplatin-related vascular pain using objective evaluation methods^3^.

Pain is difficult to quantify because the patient’s pain threshold can be influenced by their mental condition^11^. PainVision PS-2100 (Nipro Co., Osaka, Japan) is a device designed to assess patients’ sensory nociception, including perception, quantitatively and objectively, by measuring the stimulating electric current^12–14^. PainVision can evaluate pain within a few minutes without causing additional pain to the patients. In this study, we aimed to assess oxaliplatin-induced vascular pain using the PainVision system and to clarify the relationship between vessel dimeter and vascular pain in patients with CRC.

## Results

A total of 98 patients received chemotherapy for metastatic CRC between April and September 2014. The cohort included 57 men and 41 women, aged 33–87 years (median age, 67 years). The demographics and characteristics of the study patients are shown in Table 1.

**Table 1:** Demographics and characteristics of patients with colorectal cancer receiving chemotherapy.

	Oxaliplatin (+) n = 78	Oxaliplatin (−) n = 20	*P*-value
Age (years)^§^	66 (37–83)	69.5 (33–87)	0.498^¶^
Sex ratio(M:F)	43: 35	14: 6	0.311^#^
**Chemotherapy**			
CAPOX + BV	61		
CAPOX	12		
SOX	5		
XELIRI + BV		6	
SIRB		6	
Capecitabine + BV		8	
**Puncture site**			
Median cubital vein: Median vein of forearm	39: 39	9: 11	0.804^#^
Vessel diameter^*^(cm)	3.6 (1.58)	3.3 (1.21)	0.516^¶^

^§^Median (interquartile range). ^*^Mean (SD). ^¶^Unpaired Student’s *t*-test and ^#^Fisher’s exact test.

CAPOX: oxaliplatin and capecitabine; BV: bevacizumab; SOX: oxaliplatin and tegafur, gimeracil, oteracil potassium; XELIRI: irinotecan and capecitabine; SIRB: irinotecan and tegafur, gimeracil, oteracil potassium.

Seventy-eight patients (43 male, 35 female) received chemotherapy including peripheral venous oxaliplatin. The venipuncture site was the median cubital vein in 39 and the median vein of the forearm in 39.

Twenty patients (14 male, 6 female) received chemotherapy without oxaliplatin, including 12 patients who received irinotecan and eight who received neither irinotecan nor oxaliplatin. The venipuncture sites were the median cubital vein in nine and the median vein of the forearm in 11. There was no significant difference between the groups in terms of gender or venipuncture site.

### Effect of regimen on VAS

**Figure 1:**
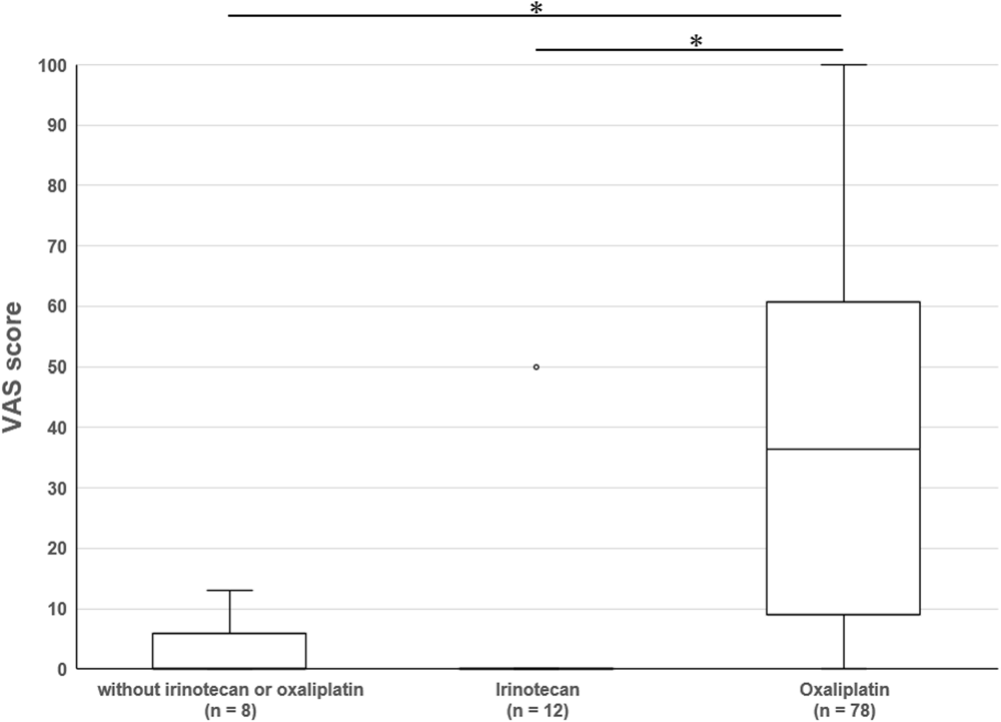
Effect of chemotherapy regimen on VAS. Vascular pain assessed by VAS score was compared among patients with CRC receiving chemotherapy including neither irinotecan nor oxaliplatin (n = 8); irinotecan (n = 12); or oxaliplatin (n = 78) (Kruskal–Wallis test, *P* < 0.001). Pairwise comparison showed that the VAS score was significantly higher in patients receiving oxaliplatin compared with those without irinotecan or oxaliplatin or with irinotecan but without oxaliplatin (*P* < 0.001). A family-wise error rate was controlled by the closed testing procedure. ^*^P < 0.001 by Kruskal–Wallis test.

The median (interquartile range) VAS scores and vascular pain in patients without irinotecan and oxaliplatin, with irinotecan, and with oxaliplatin were 0.0 (0–4.0), 0.0 (0–0), and 36.5 (9.0–60.0), respectively (*P* < 0.001 by Kruskal–Wallis test for the three groups comparison). Pairwise comparison of the three groups showed no significant difference in VAS scores between patients without irinotecan and oxaliplatin and those with irinotecan (*P* = 0.385), but the VAS score was significantly higher in patients receiving oxaliplatin compared with those without oxaliplatin (*P* < 0.001) (Fig. 1).

### Effect of gender on VAS

**Figure 2:**
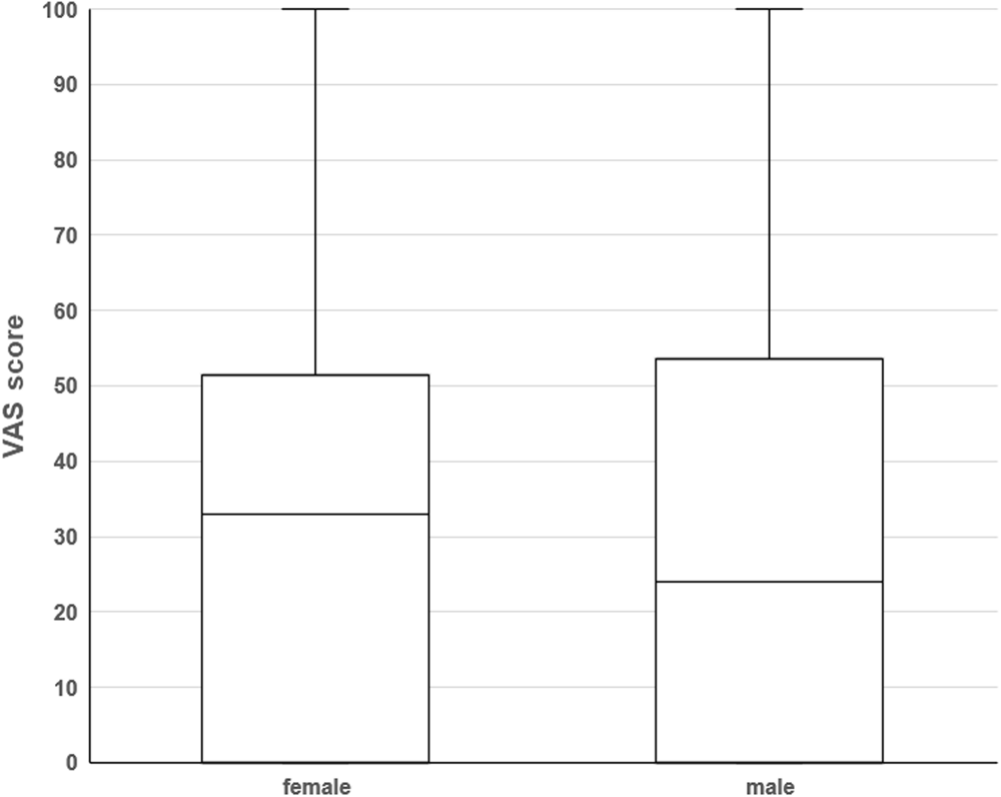
Effect of gender on VAS score. The 98 patients receiving peripheral venous chemotherapy with or without oxaliplatin for CRC included 41 females and 57 males. There was no significant difference in VAS scores between males and females (Wilcoxon’s test, *P* = 0.767).

We compared VAS scores between genders for each type of chemotherapy. Among the 41 female patients, 35 received oxaliplatin and the median female VAS score was 33 (0.0–51.0). Forty-three of the 57 male patients received oxaliplatin, and the median male VAS score was 24 (0.0–53.0). There was no significant difference in VAS scores between male and female patients with CRC administered peripheral venous chemotherapy (Wilcoxon test, *P* = 0.767) (Fig. 2).

### Correlations between VAS score and pain intensity, vessel diameter, and age

**Figure 3:**
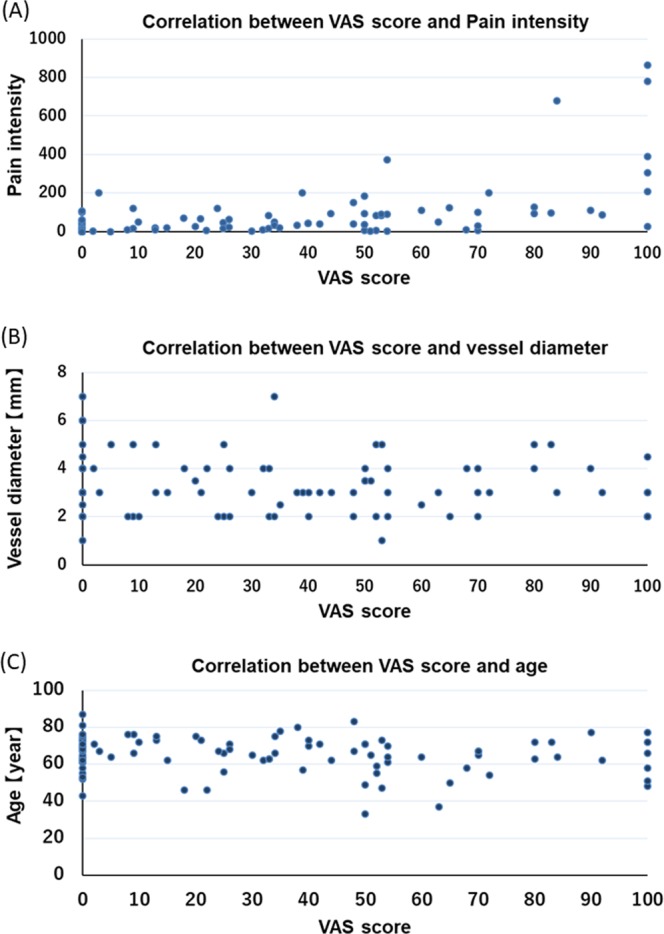
Correlations between VAS and Pain intensity, vessel diameter, and age. The 98 patients receiving peripheral venous chemotherapy with or without oxaliplatin for CRC. (**A**) Correlation between VAS score and Pain intensity (r = 0.584, *P* =< 0.001). (**B**) Correlation between VAS and vessel diameter (r = −0.107, *P* = 0.296). (**C**) Correlation between VAS and age (r = −0.174, *P* = 0.086).

We investigated the correlations between VAS score and pain intensity, vessel diameter, and age during chemotherapy administration using Spearman’s correlation coefficient. The mean VAS score and pain intensity for vascular pain were 32.0 (32.8) and 79.6 (144.6), respectively (r = 0.584, *P* < 0.001) (Fig. 3A).

The mean of age and vessel diameter were 67 years (33–87) and 3.3 mm (1–7), respectively.

There was no correlation between VAS score and age or vessel diameter (r = −0.174, *P* = 0.086, and r = −0.107, *P* = 0.296, respectively) (Fig. 3B,C).

## Discussion

We previously reported on the subjective and objective assessment of oxaliplatin-induced peripheral neuropathy^15^. However, no studies have reported on the evaluation of vascular pain during peripheral venous administration of chemotherapy, with or without oxaliplatin in patients with CRC. It is possible to distinguish between vascular pain and acute neuropathy in that the former is asymmetrical and originates at a puncture site, whereas the latter is symmetrical and occurs at the tips of the extremities. Assessment of pain can vary greatly depending on the mood and physical state of the patient at the time of assessment; thus, it is best to objectively evaluate pain. Pain evaluation by VAS is associated with a margin of error of approximately ±20 mm^10^. Therefore, a method for objective assessment is also required when evaluating drugs designed to ameliorate vascular pain. We assessed subjective pain by VAS and objective pain using the PainVision device. VAS and PainVision were both fast and easy systems to apply. The results indicated that peripheral venous administration of oxaliplatin increased the VAS score, which correlated with the Pain intensity. This is mainly attributable to the relationship between VAS scores >8 and high pain intensities.

Co-infusion of dexamethasone^7^, prewarming peripheral blood vessels^16^, premedication with oxycodone, and altering the dose rate have all been investigated as countermeasures against oxaliplatin-induced vascular pain^6^. The effects of many of these countermeasures on vascular pain were assessed by VAS. However, we considered that the additional use of the PainVision system would also allow the efficacies of these countermeasures to be assessed more objectively.

Heating the blood vessels is thought to reduce vascular pain^16^. Furthermore, blood flow is known to increase with increasing vessel diameter^17^, and Hagen Poiseuille’s equation showed that blood flow increased in proportion to the fourth power of the vessel diameter^18^. It is possible that an increase in blood flow may decrease the osmotic pressure and reduce the contact time between the anticancer drug and the vascular endothelium. We therefore examined the relationship between the VAS score for vascular pain and blood vessel diameter in patients received peripheral venous chemotherapy, but found no significant correlation.

Sex-related differences in the experience of both clinical and experimentally induced pain have been widely reported^19^, while pain sensitivity is also thought to decrease with increasing age. However, the current study found no significant differences in VAS scores in relation to either gender or age.

Although the results of the current study provide valuable information on oxaliplatin-induced vascular pain, the frequency with which patient-reported vascular pain occurs remains unclear. Further studies are needed to clarify its causes and possible countermeasures. It is also necessary to assess countermeasures against vascular pain based on objective and subjective evaluations. We are currently conducting a clinical trial to establish and validate new hypotheses regarding the relationship between vessel diameter and vascular pain. On the basis of our findings and unpublished data, we have developed a system for alleviating vascular pain. Objective evaluation of pain is necessary to accurately evaluate this system and we are investigating a means of achiving this using PainVision and VeinViewer (Luminex, Memphis, TN, USA). We plan to publish our findings in as timely a manner as possible.

## Conclusion

This study confirmed that peripheral venous administration of oxaliplatin chemotherapy induces vascular pain in patients with CRC, irrespective of blood vessel diameter.

## Methods

### Study design

This single-center study was performed at Fukuoka University Hospital between April 2014 and September 2014. Ninety-eight patients with histologically proven metastatic CRC were enrolled in this study. We only included patients with CRC in this analysis because this is the only condition for which oxaliplatin may be prescribed in Japan. Patients who did and did not receive oxaliplatin were evaluated. This evaluation was carried out immediately after the first infusion to exclude the effects of oxaliplatin-induced neuropathy. Patients with poor performance status and mental health who were unable to undergo PainVision and VAS assessments were excluded. Patients unable to feel pain due to peripheral sensory neuropathy or musculoskeletal pain were also excluded. Informed consent was obtained from all patients prior to study entry. This study was approved by Fukuoka University Hospital’s Institutional Review Board (No. 13-4-07). All procedures were performed in accordance with the Declaration of Helsinki.

### Chemotherapy

Seventy-eight patients received oxaliplatin-based chemotherapy, including 61 who received CAPOX plus bevacizumab therapy (7.5 mg/kg bevacizumab and 130 mg/m^2^ oxaliplatin on day 1 and 2000 mg/m^2^ capecitabine on days 1–14, every 3 weeks), 12 who received CAPOX therapy (130 mg/m^2^ oxaliplatin on day 1 and 2000 mg/m^2^ capecitabine on days 1–14, every 3 weeks),and five who received SOX therapy (130 mg/m^2^ oxaliplatin on day 1 and 80 mg/m^2^ tegafur, gimeracil, oteracil potassium on days 1–14, every 3 weeks).

Twenty patients received non-oxaliplatin-based chemotherapy, including eight who received capecitabine plus bevacizumab (7.5 mg/kg bevacizumab on day 1 and 2000 mg/m^2^ capecitabine on days 1–14, every 3 weeks), six who received SIRB therapy (7.5 mg/kg bevacizumab and 150 mg/m^2^ irinotecan on day 1 and 40–60 mg/body S-1 on days 1–14, every 3 weeks), and six who received XELIRI therapy (250 mg/m^2^ irinotecan on day 1 and 2000 mg/m^2^ capecitabine on days 1–14, every 3 weeks).

Vessel diameter was measured immediately before venipuncture using a tourniquet. Pain intensity after infusion of chemotherapy was measured by VAS and PainVision.

### PainVision PS-2100

The PainVision system can measure pain intensity objectively^3,15^. The system quantifies pain intensity based on the detection of electrical stimulation. Pain intensity was measured by PainVision; however, the length of the affected vessel in which pain was experienced was not measured. All measurements such as vessel diameter and PainVision were performed by the same investigator to maximize reproducibility. A disposable electrode EL-BAND was attached to the patient’s arm on the opposite side to the anticancer drug administration. A pulse-shaped current wave, which did not cause pain to the skin, was then applied and the magnitude of pain and stimulation detection were compared while gradually increasing the level of stimulation (50 Hz; 0–150 µA rms; pulse width: 0.3 ms). The patient was required to push the stop button when they first detected the electric stimulation and the current at this point was defined as the minimum perceived current. This procedure was repeated three times and the minimum sense current value (current perception threshold) was obtained from the average value. The minimum perceived current was recorded as the current perception threshold. The sensitivity of the nerve can be evaluated based on the current perception threshold. The patient was then asked to push the stop button when they considered that the current stimulus was equal to the oxaliplatin-induced vascular pain, and this current was defined as the pain-equivalent current. This measurement was also repeated three times to obtain the average pain-equivalent current value. Pain intensity was then calculated as follows:

$$\begin{array}{ccc}{\rm{Pain}}\,{\rm{intensity}} & = & (\mathrm{pain} \mbox{-} \mathrm{equivalent}\,{\rm{current}}-{\rm{minimum}}\,{\rm{perceived}}\,{\rm{current}})\\  &  & /{\rm{minimum}}\,{\rm{perceived}}\,{\rm{current}}\times 100.\end{array}$$

### Statistical analyses

Because this was an exploratory observational study, vascular pain was assessed in all patients with CRC who underwent chemotherapy within the study period. Given that no data were available to enable this, we were not able to calculate the required sample size. Data were collected and analyzed using SAS version 9.3 (SAS Institute, Cary, NC, USA). We investigated the reliability of the PainVision device in terms of its internal consistency by quantitatively assessing vascular pain using the VAS and PainVision system twice each. Data are reported as mean ± standard deviation (SD), median (interquartile range 25–75%), or number of participants (percentage).

Three groups were compared by Kruskal-Wallis test. Pairwise comparison of the three groups was performed only if Kruskal-Wallis test was significant. A family-wise error rate was controlled by the closed testing procedure.

The relationship between vascular pain (VAS score and Pain intensity) and vessel diameter after adjusting for sex and subject was assessed by partial correlation analysis^20^. Specifically, the correlation coefficient between Pain intensity and VAS score was computed using residual values of the mixed-effects model including sex as a fixed effect and subject as a random effect. A *P* value < 0.05 was considered statistically significant.
